# A Case of Advanced Biliary Tract Cancer With *EGFR* Amplification That Responded to Necitumumab

**DOI:** 10.1002/cnr2.70053

**Published:** 2024-11-14

**Authors:** Makoto Sugimori, Masaki Nishimura, Kazuya Sugimori, Sho Tsuyuki, Akane Hirotani, Haruo Miwa, Takashi Kaneko, Haruka Hirose, Yoshiaki Inayama, Akito Nozaki, Kazushi Numata, Chikara Kunisaki, Shin Maeda

**Affiliations:** ^1^ Division of Cancer Genome Medicine Yokohama City University Medical Center Yokohama Japan; ^2^ Gastroenterological Center Yokohama City University Medical Center Yokohama Japan; ^3^ Division of Genomics Laboratory Yokohama City University Medical Center Yokohama Japan; ^4^ Division of Diagnostic Pathology Yokohama City University Medical Center Yokohama Japan; ^5^ Department of Surgery Gastroenterological Center, Yokohama City University Medical Center Yokohama Japan; ^6^ Department of Gastroenterology Yokohama City University Graduate School of Medicine Yokohama Japan

**Keywords:** biliary tract cancer, case report, drug resistance, *EGFR*, liquid biopsy

## Abstract

**Background:**

Recent advances in cancer genome analysis and the practice of precision medicine have made it possible to identify fractions with rare genetic alterations. Among biliary tract cancers, *EGFR*‐amplified cancers are known to be rare fractions across organs and have a poor prognosis. The use of anti‐EGFR antibody for *EGFR*‐amplified cancers has been promising; however, the evidence is not yet clear.

**Case:**

In this report, we describe the case of a 48‐year‐old man diagnosed with advanced gallbladder cancer. The patient was administered gemcitabine plus cisplatin, followed by S‐1 monotherapy; however, disease progression was observed after two cycles of each regimen. Comprehensive genomic profiling test revealed *EGFR*‐amplification, and the patient was treated with combination therapy with the anti‐EGFR antibody necitumumab, gemcitabine, and cisplatin. After two cycles of treatment, tumor size reduced, and the treatment response was evaluated as partial response. On Day 90, after five cycles of treatment, tumor progression was confirmed. In addition, after disease progression, liquid biopsy revealed acquired pathogenic gene alterations suggesting anti‐EGFR antibody resistance.

**Conclusion:**

This report supports the clinical benefit of anti‐EGFR antibodies for *EGFR*‐amplified biliary tract cancers and the importance of genomic analysis in personalized therapy and drug resistance research.

## 1 Introduction

Biliary tract cancer (BTC) is a relatively rare cancer, with an estimated incidence of fewer than six cases per 100 000 population; however, its incidence is increasing worldwide. In addition, BTC is a highly refractory cancer with one of the worst prognoses. Due in part to its anatomic complexity, early diagnosis is difficult; most patients present with locally advanced or metastatic disease at the time of diagnosis [1, 2]. Genomic profiling of BTC has revealed a variety of driver genes, and the practice of precision medicine is required [3]. Although effective molecular‐targeted drugs available for human use remain limited, anti‐EGFR antibodies have been clinically applied to cancer types that originally express high levels of *EGFR*. Theoretically, anti‐EGFR antibodies are expected to be effective against *EGFR*‐amplified cancer, and there are basic and clinical studies suggesting the utility [4, 5].

Biliary tissue is reported to express relatively high levels of *EGFR*, and the efficacy of anti‐EGFR antibodies against BTC has been evaluated [6]. However, all clinical trials failed to demonstrate the benefit of adding anti‐EGFR antibodies to gemcitabine (GEM)‐based regimens for advanced BTC without enrichment due to *EGFR* amplification [7].

Here, we describe a case of advanced *EGFR*‐amplified BTC that responded to combination therapy with anti‐EGFR antibody necitumumab, GEM, and cisplatin (CDDP). After treatment, acquired pathogenic gene alterations in *EGFR*, *KRAS*, *RET*, *CTNNB1*, and *FH* were detected by liquid biopsy.

## 2 Case Presentation

**FIGURE 1:**
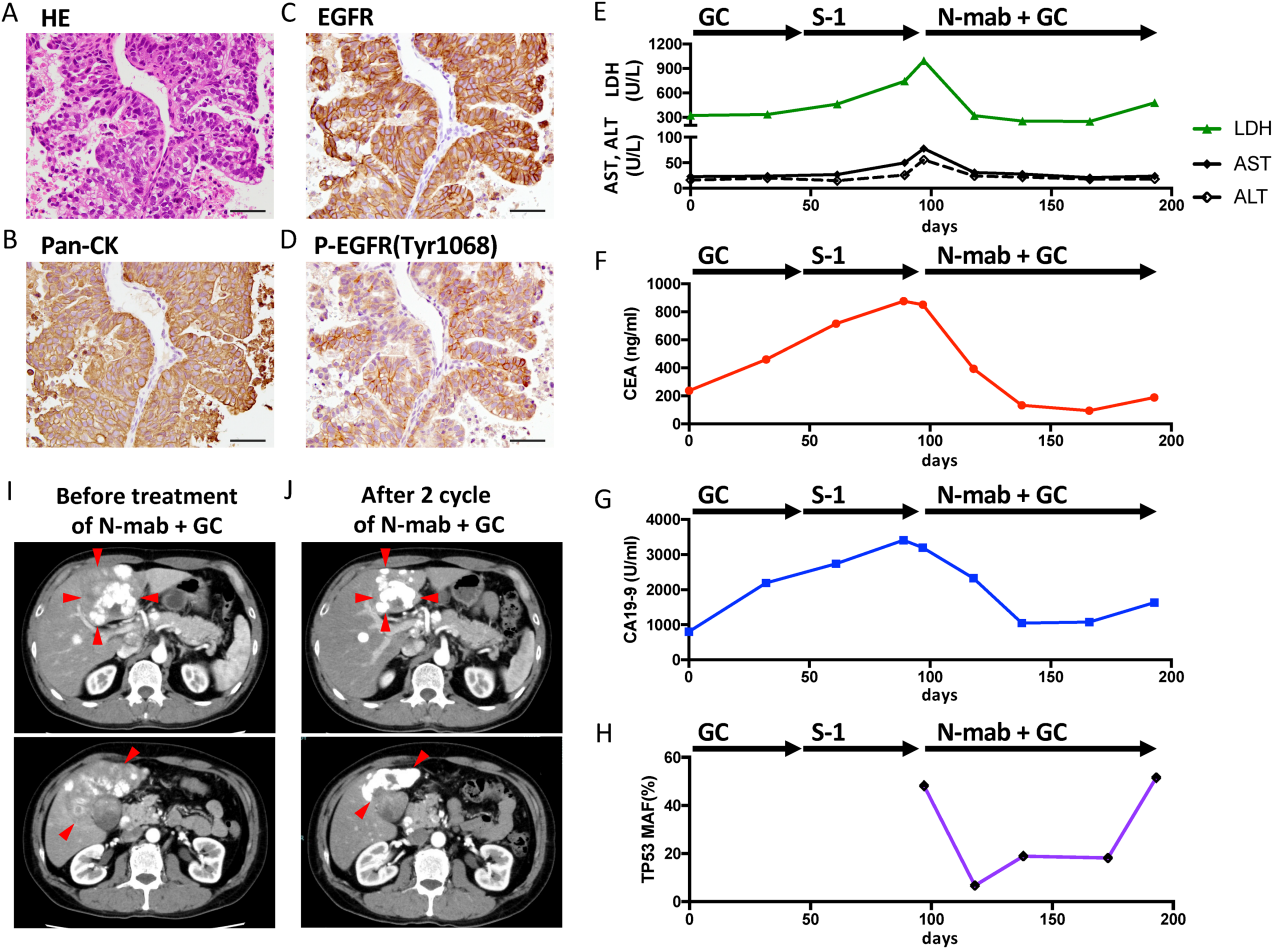
Clinical presentation. Representative pathology slides for liver biopsy specimen (A–D; scale bars represent 50 μm, 400‐fold magnification). (A) Hematoxylin and eosin, (B) pan‐cytokeratin, (C) *EGFR*, (D) phospho‐EGFR Tyr1068. Course of informative markers (E–H). (E) Aspartate transaminase (AST), alanine transaminase (ALT), and lactate dehydrogenase (LDH), (F) carcinoembryonic antigen (CEA), (G) carbohydrate antigen 19‐9 (CA19‐9), (H) *TP53* C135fs*35 MAF in cell‐free DNA. Contrast‐enhanced CT image of the abdomen (I, J). (I) Before (baseline); (J) after 2 cycles of necitumumab + GC treatment. Arrowheads indicate the margin of the tumor. MAF, mutation allele frequency; N‐mab, necitumumab; GC, gemcitabine + cisplatin.

A 48‐year‐old man presenting with a chief complaint of epigastric pain was referred to Yokohama City University Medical Center in March of 2022. After imaging tests and tumor biopsy, the patient was diagnosed with gallbladder cancer with multiple liver and lymph node metastases (Figure 1A,B). He was administered GEM (1000 mg/m^2^) plus CDDP (25 mg/m^2^), followed by S‐1 (120 mg/body) monotherapy; however, disease progression was observed after two cycles of each regimen. A comprehensive genomic profiling test (CGP) was performed on an archival biopsy sample using the NCC Oncopanel (Sysmex Corporation, Hyogo, Japan) [8]. CGP revealed the amplification of *EGFR* and mutation of *TP53* C135fs*35 and *CDKN2A* R80* (Table 1, panel A). Immunohistochemical analysis showed strong staining for EGFR and phosphorylated EGFR (Figure 1C,D, Data S1). Based on CGP, the patient was treated with necitumumab, GEM, and CDDP (necitumumab + GC) as an off‐label therapy (3‐week cycle, Day1 and 8; necitumumab [800 mg] and GEM [1000 mg/m^2^], Day1; CDDP [25 mg/m^2^]). After the initiation of the treatment, liver enzyme, lactate dehydrogenase, and tumor marker levels decreased significantly (Figure 1E–G). The value of the *TP53* C135fs*35 mutation allele frequency (MAF) in cell‐free DNA (cf‐DNA) was quantified by droplet digital PCR (Bio‐Rad, CA, USA), showing a marked decrease (Figure 1H, Data S1). Contrast‐enhanced CT after two cycles of necitumumab + GC treatment showed a reduction in tumor size, and the treatment response was evaluated as partial response (Figure 1I,J). During treatment, grade 1 skin rash appeared after the first course, which could be controlled with topical steroid. Grade 3 neutropenia occurred during the third cycle, and the GEM dose was reduced (800 mg/m^2^). On Day 90, after five cycles of necitumumab + GC treatment, tumor progression was confirmed. After necitumumab + GC treatment, FoundationOne Liquid CDx (F1LCDx; Foundation Medicine, Boston, MA, USA) [8] revealed the emergence of pathogenic gene alterations in *EGFR*, *KRAS*, *RET*, *CTNNB1*, and *FH* (Table 1, panel B).

**TABLE 1:** Summary of the detected pathogenic gene alterations.

(A) NCC Oncopanel	
Gene	Amplification, copy number
*EGFR*	42.60
*PRKCI*	12.14
Gene	Mutation, amino acid replacement (mutation allele frequency [%])
*TP53*	C135fs*35 (68.50)
*CDKN2A*	R80* (63.30)

(B) FoundationOne Liquid CDx	
Gene	Amplification, copy number
*EGFR*	6.96
*PRKCI*	5.03
*TERC*	4.63
*FGF10*	2.48
Gene	Mutation, amino acid replacement (mutation allele frequency [%])
*TP53*	C135fs*35 (48.23)
*CDKN2A*	R80* (28.37)
*CTNNB1*	G34R (0.32), T41I (0.13), S33C (0.21), S33A (0.5), S33F (0.43), D32V (0.33), S33P (0.61), S29F (0.13), S37F (0.28), D32G (1.08), D32H (0.52)
*EGFR*	S229C (0.07), G719A (0.07)
*KRAS*	Q61H (6.62, 0.37)^a^
Gene	Rearrangement, description (mutation allele frequency [%], read count)
*ASXL1*	*ASXL1* (NM_015338) inversion intron 6–intron 11 (4.01, 52)
*RET*	*CCDC6* (NM_005436)‐*RET*(NM_020630) fusion (C1; R12) (1.88, 91)
*EGFR*	*EGFR* (NM_005228) deletion intron 1–intron 7 (*EGFR* vIII) (0.66, 136) *EGFR* (NM_005228) inversion intron 1–intron 7 (0.33, 104) *EGFR* (NM_005228) duplication intron 1–intron 7 (0.23, 49) *EGFR* (NM_005228) rearrangement intron 26 (0.23, 20) *EGFR* (NM_005228) deletion intron 13–intron 15 (0.14, 53) *EGFR* (NM_005228) rearrangement intron 25 (0.04, 12) *EGFR* (NM_005228) duplication intron 17–exon 27 (0.03, 61) *EGFR* (NM_005228) rearrangement exon 27 (0.03, 13) *SEC61G* (NM_014302)‐*EGFR*(NM_005228) fusion (S2; E13) (0.46, 206)
*FH*	*FH* (NM_000143) rearrangement exon 5 (5.88, 84)

Abbreviation: *EGFR* vIII, *EGFR* variant III.

^a^ Different nucleotide variants with the same amino acid.

## 3 Published Data Review

**FIGURE 2:**
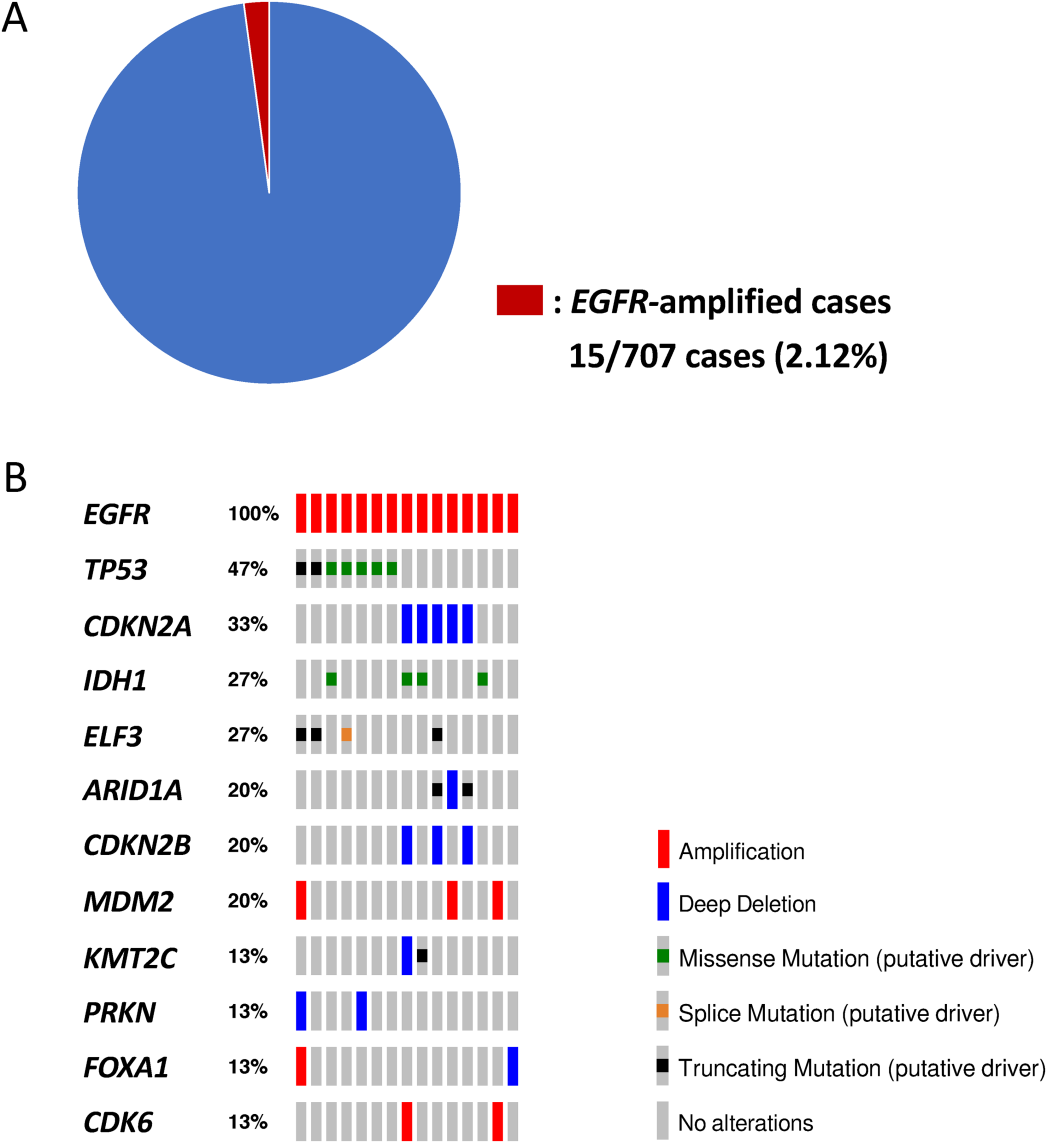
Genomic features of *EGFR*‐amplified biliary tract cancer in the dataset of 707 previously reported cancers, available at c‐BioPortal (https://www.cbioportal.org/) [9, 10, 11, 12]. (A) Proportion of *EGFR*‐amplified cases. (B) Oncoprot of 15 *EGFR*‐amplified cases. Gene alterations were annotated using OncoKB (https://www.oncokb.org).

To evaluate the prevalence and genomic profile of *EGFR*‐amplified BTC, we reviewed the previously reported tissue‐genome sequence datasets of 707 BTC samples (51 samples of cholangiocarcinoma [Firehose Legacy, TCGA], 412 samples of intrahepatic cholangiocarcinoma, and 244 samples of gallbladder cancer), available at c‐BioPortal (https://www.cbioportal.org/) [9, 10, 11, 12]. *EGFR* amplification was identified in 15/707 samples (2.12%), and the most common co‐existing pathogenic gene alterations included *TP53* (47%) and *CDKN2A* (33%) (Figure 2A,B).

## 4 Discussion

The recent practice of precision medicine has made it possible to identify rare fractions. We encountered a case of *EGFR*‐amplified BTC and reviewed 707 publicly available BTC datasets. We found that [1] *EGFR*‐amplified cases are extremely rare (2.12%), and [2] the coexistence of pathogenic mutations in *TP53* and *CDKN2A* observed in the present case is a typical pattern (Table 1, Figure 2).

Of the anti‐EGFR antibodies in clinical use, necitumumab is the only IgG1 human monoclonal antibody, and it has advantages of safety and therapeutic benefits such as low risk of infusion reactions and long half‐life [13]. Further, IgG1 antibodies have high antibody‐dependent and complement‐dependent cytotoxicity, and are expected to have antitumor effects [13]. Necitumumab + GC therapy is already being clinically applied to lung cancer, [14] and we used this therapy in the present case. Tumor size reduction was observed after two cycles of necitumumab + GC treatment; hence, the effect of add‐on necitumumab to GC is evident, compared with the clinical course of the first‐line GC treatment. During the treatment, tumor markers, as well as *TP53* C135fs*35 MAF values in cf‐DNA, showed a correlation with disease status. Quantitative monitoring of cf‐DNA was reported to be useful for assessing disease status, and its utility was also demonstrated [15, 16].

The timing of chemotherapy resistance is dramatic, and elucidating the mechanisms is critical. F1LCDx performed after tumor progression detected various pathogenic gene alterations (Table 1, panel B). Interestingly, numerous variants were detected in *EGFR*, especially in the extracellular domain (ECD), such as *EGFR* variant III (Table 1, panel B, Figure S1). Alterations in the ECD of *EGFR* are known to prevent the binding of anti‐EGFR antibodies [17, 18, 19]. Additional gene alterations detected were *KRAS* mutation and *RET* fusion, which activate RTK pathways downstream of *EGFR*; *CTNNB1* mutation, which activates the Wnt pathway; and loss of *FH*, which causes oncogenic metabolic change. These findings suggest the underlying basis of anti‐EGFR antibody resistance and tumor progression. However, the limitation of this report is that CGP performed before and after treatment were not identical. Therefore, the presence of these pathogenic variants prior to treatment cannot be completely ruled out.

Anti‐EGFR antibodies have been clinically applied to colorectal cancer. Although *EGFR*‐amplified colorectal cancer is also rare (1%), it has been reported to be associated with a good prognosis because of the benefits of anti‐EGFR antibodies [20]. Treatment of *EGFR*‐amplified solid tumors remains an unmet medical need, and therapeutic strategies urgently need to be developed. To the best of our knowledge, this is the first report suggesting the therapeutic efficacy of necitumumab + GC therapy in *EGFR*‐amplified BTC. Based on this report, we have initiated a phase II study to investigate the efficacy of necitumumab and GEM combination therapy for *EGFR*‐amplified BTC (jRCTs031230259).

## 5 Conclusions

In this report, we describe a case of advanced *EGFR*‐amplified gallbladder cancer that responded to combination therapy with the anti‐EGFR antibody necitumumab, GEM, and CDDP. In addition, we used liquid biopsy to monitor the development of resistance and elucidate the molecular mechanism of resistance. A review of the published data indicates that BTCs with *EGFR*‐amplification are a rare fraction (accounting for about 2.1%); however, effective utilization of existing drugs is a pressing issue. Hence, treatment with anti‐EGFR antibodies for *EGFR*‐amplified BTCs should be validated by further studies.

## Author Contributions

**Makoto Sugimori:** conceptualization, methodology, data curation, writing – original draft, project administration. **Masaki Nishimura:** conceptualization, writing – original draft, methodology, project administration, data curation. **Kazuya Sugimori:** conceptualization, writing – review and editing. **Sho Tsuyuki:** conceptualization, writing – review and editing. **Akane Hirotani:** conceptualization, writing – review and editing. **Haruo Miwa:** conceptualization, writing – review and editing. **Takashi Kaneko:** conceptualization, writing – review and editing. **Haruka Hirose:** data curation, methodology, project administration. **Yoshiaki Inayama:** methodology, data curation, project administration. **Akito Nozaki:** conceptualization, project administration, supervision, writing – review and editing. **Kazushi Numata:** conceptualization, writing – review and editing, project administration, supervision. **Chikara Kunisaki:** conceptualization, writing – review and editing, project administration, supervision. **Shin Maeda:** conceptualization, writing – review and editing, project administration, supervision.

## Ethics Statement

The off‐label use of the drug limited to this patient was approved by the Institutional Review Board of Yokohama City University Medical Center. Analysis of the cf‐DNA was approved by the Regional Committee for Medical and Health Research Ethics of Yokohama City University (approval number: B160804006), and the patient was provided informed consent prior to the specimen collection.

## Consent

Informed consent for publication of this case report has been obtained from the patient.

## Conflicts of Interest

The authors declare no conflicts of interest.

## Supporting information


Figure S1.

Data S1.

## Data Availability

Data sharing not applicable to this article as no datasets were generated or analyzed during the current study.

## References

[1] J. M. Banales , J. J. G. Marin , A. Lamarca , et al., “Cholangiocarcinoma 2020: The Next Horizon in Mechanisms and Management,” Nature Reviews. Gastroenterology & Hepatology 17, no. 9 (2020): 557–588. 32606456 10.1038/s41575-020-0310-z PMC7447603

[2] D. Kim , P. Konyn , G. Cholankeril , C. A. Bonham , and A. Ahmed , “Trends in the Mortality of Biliary Tract Cancers Based on Their Anatomical Site in the United States From 2009 to 2018,” American Journal of Gastroenterology 116, no. 5 (2021): 1053–1062. 33929380 10.14309/ajg.0000000000001151

[3] T. B. Karasic , J. R. Eads , and L. Goyal , “Precision Medicine and Immunotherapy Have Arrived for Cholangiocarcinoma: An Overview of Recent Approvals and Ongoing Clinical Trials,” JCO Precision Oncology 7 (2023): e2200573. 37053534 10.1200/PO.22.00573 PMC10309532

[4] Y. Nakamura , A. Sasaki , H. Yukami , et al., “Emergence of Concurrent Multiple EGFR Mutations and MET Amplification in a Patient With EGFR‐Amplified Advanced Gastric Cancer Treated With Cetuximab,” JCO Precision Oncology 4 (2020). 10.1200/PO.20.00263 PMC7713584 33283138

[5] S. Kato , R. Okamura , M. Mareboina , et al., “Revisiting Epidermal Growth Factor Receptor (EGFR) Amplification as a Target for Anti‐EGFR Therapy: Analysis of Cell‐Free Circulating Tumor DNA in Patients With Advanced Malignancies,” JCO Precision Oncology 3 (2019). 10.1200/PO.18.00180 PMC6497417 31058253

[6] T. Kawamoto , K. Ishige , M. Thomas , et al., “Overexpression and Gene Amplification of EGFR, HER2, and HER3 in Biliary Tract Carcinomas, and the Possibility for Therapy With the HER2‐Targeting Antibody Pertuzumab,” Journal of Gastroenterology 50, no. 4 (2015): 467–479. 25112701 10.1007/s00535-014-0984-5

[7] A. Rizzo , G. Frega , A. D. Ricci , et al., “Anti‐EGFR Monoclonal Antibodies in Advanced Biliary Tract Cancer: A Systematic Review and Meta‐Analysis,” In Vivo 34, no. 2 (2020): 479–488. 32111744 10.21873/invivo.11798 PMC7157865

[8] H. Ebi and H. Bando , “Precision Oncology and the Universal Health Coverage System in Japan,” JCO Precision Oncology 3 (2019). 10.1200/PO.19.00291 PMC7446489 32923862

[9] T. Boerner , E. Drill , L. M. Pak , et al., “Genetic Determinants of Outcome in Intrahepatic Cholangiocarcinoma,” Hepatology 74, no. 3 (2021): 1429–1444. 33765338 10.1002/hep.31829 PMC8713028

[10] N. A. Giraldo , E. Drill , B. A. Satravada , et al., “Comprehensive Molecular Characterization of Gallbladder Carcinoma and Potential Targets for Intervention,” Clinical Cancer Research 28, no. 24 (2022): 5359–5367. 36228155 10.1158/1078-0432.CCR-22-1954 PMC9772093

[11] E. Cerami , J. Gao , U. Dogrusoz , et al., “The cBio Cancer Genomics Portal: An Open Platform for Exploring Multidimensional Cancer Genomics Data,” Cancer Discovery 2, no. 5 (2012): 401–404. 22588877 10.1158/2159-8290.CD-12-0095 PMC3956037

[12] J. Gao , B. A. Aksoy , U. Dogrusoz , et al., “Integrative Analysis of Complex Cancer Genomics and Clinical Profiles Using the cBioPortal,” Science Signaling 6(269):pl1 (2013). 10.1126/scisignal.2004088 PMC4160307 23550210

[13] R. Dienstmann and J. Tabernero , “Necitumumab, a Fully Human IgG1 mAb Directed Against the EGFR for the Potential Treatment of Cancer,” Current Opinion in Investigational Drugs 11, no. 12 (2010): 1434–1441. 21154125

[14] N. Thatcher , F. R. Hirsch , A. V. Luft , et al., “Necitumumab Plus Gemcitabine and Cisplatin Versus Gemcitabine and Cisplatin Alone as First‐Line Therapy in Patients With Stage IV Squamous Non‐small‐Cell Lung Cancer (SQUIRE): An Open‐Label, Randomised, Controlled Phase 3 Trial,” Lancet Oncology 16, no. 7 (2015): 763–774. 26045340 10.1016/S1470-2045(15)00021-2

[15] M. Sugimori , K. Sugimori , H. Tsuchiya , et al., “Quantitative Monitoring of Circulating Tumor DNA in Patients With Advanced Pancreatic Cancer Undergoing Chemotherapy,” Cancer Science 111, no. 1 (2020): 266–278. 31746520 10.1111/cas.14245 PMC6942439

[16] M. G. Krebs , U. Malapelle , F. André , et al., “Practical Considerations for the Use of Circulating Tumor DNA in the Treatment of Patients With Cancer: A Narrative Review,” JAMA Oncology 8, no. 12 (2022): 1830–1839. 36264554 10.1001/jamaoncol.2022.4457

[17] H. K. Gan , A. N. Cvrljevic , and T. G. Johns , “The Epidermal Growth Factor Receptor Variant III (EGFRvIII): Where Wild Things Are Altered,” FEBS Journal 280, no. 21 (2013): 5350–5370. 23777544 10.1111/febs.12393

[18] C. R. Chong and P. A. Jänne , “The Quest to Overcome Resistance to EGFR‐Targeted Therapies in Cancer,” Nature Medicine 19, no. 11 (2013): 1389–1400. 10.1038/nm.3388 PMC4049336 24202392

[19] Z. An , O. Aksoy , T. Zheng , Q. W. Fan , and W. A. Weiss , “Epidermal Growth Factor Receptor and EGFRvIII in Glioblastoma: Signaling Pathways and Targeted Therapies,” Oncogene 37, no. 12 (2018): 1561–1575. 29321659 10.1038/s41388-017-0045-7 PMC5860944

[20] G. Randon , R. Yaeger , J. F. Hechtman , et al., “EGFR Amplification in Metastatic Colorectal Cancer,” Journal of the National Cancer Institute 113, no. 11 (2021): 1561–1569. 33825902 10.1093/jnci/djab069 PMC8562951

