# Supplementary material for: A Case of Advanced Biliary Tract Cancer With EGFR Amplification That Responded to Necitumumab
Source: Cancer Rep (Hoboken). 2024 Nov 14;7(11):e70053. doi: 10.1002/cnr2.70053 (PMC11561843; doi:10.1002/cnr2.70053)
Supplement: Supplementary file 1 — Figure S1. [file CNR2-7-e70053-s002.docx]

Figure S1

Detected pathogenic gene alterations in *EGFR* after necitumumab + GC treatment. Mutations are listed in the upper section, and rearrangements are listed in the lower section. GC, gemcitabine + cisplatin; ECD, extracellular domain; TM, transmembrane domain; TK, tyrosine kinase domain; del., deletion; inv., inversion; dup., duplication; trun., truncation.
