# Supplementary material for: A Case of Advanced Biliary Tract Cancer With EGFR Amplification That Responded to Necitumumab
Source: Cancer Rep (Hoboken). 2024 Nov 14;7(11):e70053. doi: 10.1002/cnr2.70053 (PMC11561843; doi:10.1002/cnr2.70053)
Supplement: Supplementary file 2 — Data S1. [file CNR2-7-e70053-s001.docx]

**Methods**

**Immunohistochemistry**

Standard procedures were applied for immunohistochemical examination. In brief, formalin-fixed and paraffin-embedded tissue samples were sectioned at 4-μm thickness. After deparaffinization and rehydration, slides were processed for 15 min at 100 °C in citrate buffer (pH 6.0) for heat-mediated antigen retrieval. Endogenous peroxidase was blocked with 3% hydrogen peroxide for 10 min at room temperature. Slides were then incubated with the primary antibodies overnight at 4 °C. The secondary antibody (#424151, polymer detection systems of Histofine SIMPLESTAIN MAX-PO(MULTI), Nichirei Biosciences, Tokyo, Japan) was applied for 30 min at room temperature, followed by chromogen staining (#425011, DAB Chromogen Kit, Nichirei Biosciences). Finally, slides were counterstained with hematoxylin and dehydrated for mounting. The following primary antibodies listed with the clone, catalog number, company, and dilution, were used: anti-pan-cytokeratin antibody (AE1/AE3, AE1/AE3-601-L-CE, Novocastra-Leica Biosystems, Nussloch, Germany, 1:100), anti-EGFR antibody (D38B1, #4267, Cell Signaling Technology, Danvers, MA, USA, 1:50), and anti-phospho-EGFR Tyr1068 antibody (D7A5, #3777, Cell Signaling Technology, 1:400).

**Quantitative ct-DNA monitoring**

Peripheral blood samples were collected in cell-free DNA collection tubes (#07785666001, Roche GmbH, Mannheim, Germany). Within 24 h of collection, samples were centrifuged for 10 min at 3000 rpm. The supernatant was collected and centrifuged for 3 min at 15,000 rpm. The supernatant was collected carefully and stored at −80 °C. Cell-free DNA extraction was performed by MagNA Pure 24 system (Roche), and the DNA concentration was assayed by Agilent 4150 TapeStation (Agilent Technologies, Santa Clara, CA, USA). To quantitate *TP53* C135fs*35 mutation frequency, we used the ddPCR MUT FAM+HEX Assay (#10049047, UniqueAssayID; dHsaMDS562846055, Bio-Rad, Hercules, CA, USA), which was designed in silico to detect *TP53* C135fs*35 mutation by FAM labeling and wild-type TP53 by HEX labeling, and analyzed by droplet digital PCR (QX200^TM^ Droplet Digital^TM^ PCR IVD System, Bio-Rad). PCR conditions were validated using the artificially synthesized 181 bp fragment of DNA from the human genome, including 90 bp before and after the *TP53* C135fs*35 mutation, as a positive control (gBlocks Gene Fragments, Integrated DNA Technologies, Coralville, IA, USA). The assay was performed according to the manufacturer’s instructions, and the data were analyzed using Quanta Soft software (Bio-Rad).
